# Theta Burst TMS over the Pre-SMA Improves Inhibitory Control in Gambling Disorder Subjects as Assessed with Stop Signal Task

**DOI:** 10.3390/brainsci15050448

**Published:** 2025-04-25

**Authors:** Gioele Gavazzi, Carlo Cavaliere, Marco Salvatore, Nikolaos Makris, Stefano Pallanti

**Affiliations:** 1 Department of Neuroscience, Psychology, Drug Research, Child Health, University of Florence, 50135 Florence, Italy; gioele.gavazzi@unifi.it; 2 IRCCS SDN, 80131 Naples, Italy; carlo.cavaliere@synlab.it (C.C.); marcosalvatore2.segreteria@gmail.com (M.S.); 3 Psychiatry Neuroimaging Laboratory, Department of Psychiatry, Brigham and Women’s Hospital, Harvard Medical School, Boston, MA 02115, USA; nikos@cma.mgh.harvard.edu; 4 Departments of Radiology and Psychiatry, Harvard Medical School, Boston, MA 02115, USA; 5 Departments of Radiology and Psychiatry, Massachusetts General Hospital, Charlestown, MA 02129, USA; 6 Department of Psychiatry and Health Sciences, Institute of Neurosciences, 50121 Florence, Italy

**Keywords:** inhibitory control, gambling, SST, cTBS, TMS, pre-SMA

## Abstract

Background. Inhibitory control failure represents a central trait in substance and behavioral addictions, which includes patients affected with gambling disorder (GD). In GD patients, research on this trait of the addiction cycle has primarily focused on the use of pharmacological treatments for its assessment. More recently, modification of neural activity using transcranial magnetic stimulation (TMS) has been used to explore the dimensions of GD using patient questionnaires. Methods. Herein, we evaluated the use of continuous Theta burst stimulation (cTBS) over the pre-supplementary motor area (pre-SMA) to determine if it modified inhibitory control in the stop signal task of patients affected by GD when compared with a group of healthy controls without cTBS treatment. Results. To the best of our knowledge, our study is the first to report that TMS treatment of GD patients is associated with a behavioral improvement of stop signal reaction time. Conclusion. Our results suggest that this TMS-mediated improvement in the efficiency of inhibitory control in GD patients warrants further mechanistic studies in a larger cohort to determine if can be used as a treatment modality.

## 1. Introduction

Gambling disorder (GD) is characterized by persistent, recurrent maladaptive patterns of gambling behavior and an inability to carry out certain functions in daily life (functional impairment). It has been recently included in the substance-related and addiction disorder from the DSM-5 [1]. This disorder is observed in the population, with a prevalence of 1.3–7.7% [2]. The most common alteration is in decision making, e.g., [3], reward functioning and inhibitory control [4,5].

An alteration in inhibitory control is considered the most relevant dysfunction in patients affected by GD, both in terms of the underlying neurobiological mechanism and its social relevance. An increased rate of suicide and self-harm has been observed in people with GD [6].

Although research in GD has consistently reported a deficit in inhibitory control [4,5], most of these studies have focused on pharmacological interventions, but only very few have investigated outcomes using electrical neuromodulation.

Repetitive transcranial magnetic stimulation (rTMS) has been hypothesized to modulate all altered functions in GD, e.g., decision-making, reward functioning and inhibitory control [7]. To the best of our knowledge, however, none of these studies reported behavioral measures; they were limited to assessment by self-reported questionnaires.

**Figure 1:**
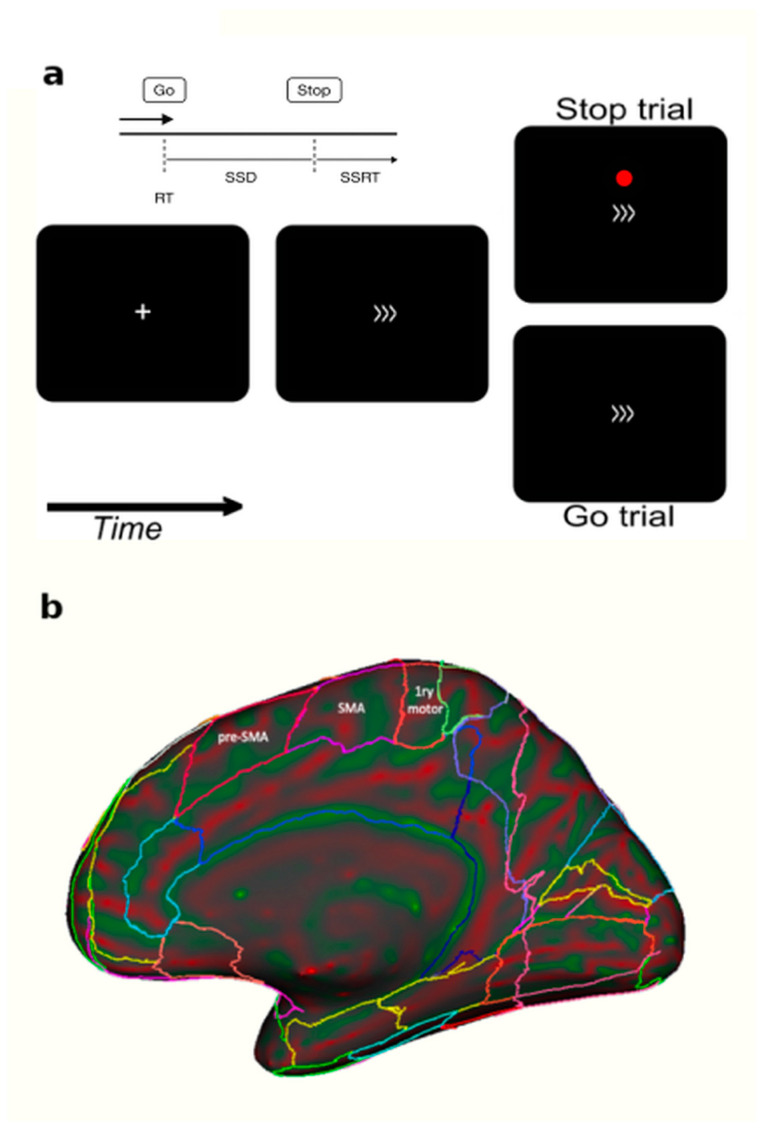
Panel (**a**) Each trial started with a fixation cross for 350 ms followed by the go stimulus for 1400 ms (a right- or left-pointing arrow)**.** Interstimulus interval ranged between 2000 to 3750 ms. Participants had to press a button when they saw a go signal (>>>) and to not press any button when a red dot was displayed. (**b**) A sagittal brain view showing the anatomical difference between pre-SMA and SMA [24]. The cTBS (TMS) or SHAM coils were applied over the pre-SMA.

The stop signal task (SST) is extensively used in basic and clinical research to study cognitive inhibitory control. From the participants’ responses, it measures their commitment to the task, their capacity to prepare for unexpected upcoming events employing proactive inhibition, and to suppress an ongoing action through reactive inhibition [8,9]. Complex designs linking more than one SST can quantify the variation in three parameters, stop signal reaction times (SSRTs), stop signal delay (SSD), and reaction time (RT), to understand the contribution of proactive inhibition. In particular, RT reflects basic motor response speed, SSD indicates the system’s tolerance before reactive inhibition is triggered, and SSRT is the key measure of the capacity to inhibit one’s responses. A schematic illustration of these parameters is shown in Figure 1a. Alone, the SSRT has proven to be a significant measure of cognitive control mechanisms involved in the inhibitory control processes. Combined with the other parameters, the SSRT can refine the characterization of the inhibitory control processes. It is well recognized that an increase in the SSD is associated with a reduction in SSRT, which suggests that strategies prior to inhibition are being implemented ostensibly through a proactive process. Under these circumstances, RT can be a perfect tool with which to assess whether an observed improvement in SSRT is due to specific task-related strategies [8,10,11,12] or whether the SSRT is due to an improvement in proactive inhibitory efficiency, e.g., [8]. A slowing of the RT makes the task easier by producing greater SSD. When there is no variation in RT between different administrations of the task, however, we know that participants are performing the task with the same commitment in all sessions. If associated with a reduction in SSRT, this increase in SSD may suggest an improved efficiency in proactive inhibition.

Several studies using the SST have shown that cognitive inhibitory control is associated with activity in brain circuitry involving the pre-supplementary motor area (pre-SMA) and right prefrontal cortices [13,14,15,16]. The application of continuous cTBS over pre-SMA in healthy subjects modulates the cognitive control [17,18]. When Obeso et al. [18] explored the effect of a session of cTBS or a SHAM over pre-SMA and right Inferior Frontal Gyrus (r-IFG) in a population of healthy subjects, they only observed a reduction of SSRT in the cTBS session. Using a small group of GD patients without mood disorder comorbidity, the use of cTBS over the pre-SMA has proven to be safe, with suggestive clinical efficacy [19,20,21], though a larger number of subjects with cognitive measures is needed to confirm efficacy and provide mechanistic insights. Thus, considering the above-reported literature, it seems that targeting pre-SMA can improve inhibitory control in populations with gambling disorders. Moreover, pre-SMA is anatomically accessible to non-invasive brain stimulation techniques and offers a practical and mechanistically grounded alternative to more traditionally targeted regions like the DLPFC and ACC [22,23].

Herein, we hypothesize that the application of cTBS over the pre-SMA will ameliorate the inhibitory processing deficit in subjects affected by GD. In this study, we assessed the inhibitory control process in a group of GD patients using SST. We have chosen this task because several studies have shown that impaired response inhibition, represented by altered SSRTs, is associated with problem gambling behavior [25,26]. We evaluated the SST at two timepoints, before and after one session of cTBS over the pre-SMA. We compared the performances of GD subjects at these timepoints to untreated controls (no cTBS) and to 2 cTBS SHAM GD Patients.

## 2. Materials and Methods

In this experiment we used SST to assess the effect of Theta burst TMS over the pre-SMA on cognitive inhibitory control in patients affected by gambling disorder, as compared with healthy controls and 2 SHAM GD patients.

### 2.1. Participants

Thirty-seven participants were enrolled in this study. Specifically, one group was composed of 18 subjects meeting criteria for GD that received TMS treatment (12 males and 6 females; mean age = 39.9 ± 11.1). Another group of 17 healthy volunteers received no TMS treatment (9 males and 8 females; mean age = 41.3 ± 14.0). A third group of two GD participants received a SHAM protocol (2 males; 37 and 35 years old). After a screening visit, the patients fulfilling the GD inclusion criteria were assessed before entering the study with a comprehensive clinical interview (GD onset, duration of the illness, previous pharmacological and psychotherapeutic treatments, treatment resistance, current and past comorbidities).

The inclusion criteria consisted of age between 18–65 years old; DSM-5 gambling disorder diagnosis; and stable pharmacological treatment for 6 weeks before enrollment.

The exclusion criteria included the presence of (1) psychiatric comorbidities, except for nicotine dependence; (2) lifetime diagnosis of schizophrenia, psychotic disorders, alcohol/drug dependence or abuse, bipolar disorder I or II, or mental disorders due to medical condition; (3) suicide risk; (4) duration of illness < 2 years; (5) hospitalization within the last 6 months before enrollment; (6) contraindications to TMS treatment; and (7) inability or unwillingness to provide written informed consent.

All participants had normal or corrected-to-normal visual acuity and normal hearing, by self-report. Written informed consent was obtained from each participant and was approved by the local ethical committee in agreement with the 1964 Helsinki declaration and its later amendments or comparable ethical standards. Finally, all patients filled out the TMS Safety Screening Questionnaire.

### 2.2. Transcranial Magnetic Stimulation Protocols

One session of repetitive TMS was administered to the GD group of patients after the resting motor threshold (RMT) was measured. We defined RMT as the minimum magnetic flux needed to elicit a threshold electromyography (EMG) response (50 mV in peak-to-peak amplitude) in a resting target muscle (abductor pollicis brevis or APB) in 5–10 trials using single-pulse TMS administered to the contralateral primary motor cortex.

Active motor threshold (AMT) was measured following the same approach during tonic contraction of hand muscles and aiming for a motor-evoked potential (MEP) greater than 200 mV in peak-to-peak amplitude.

In the current study, a continuous Theta burst stimulation (cTBS) was administered with the Magstim Rapid2 Stimulator (Magstim Company Ltd., Whitland Industrial Estate Spring Gardens, Whitland Carmarthenshire, UK) using a 70 mm, 8-shaped coil. This coil is made with 2 identical configurations for TMS (A) and SHAM (B) protocols; the technicians were blind to the use of coils “A or B”. The cTBS consisted of bursts of 3 pulses separated by 20 ms (50 Hz), with each triplet repeated every 200 ms (5 Hz). Stimulus intensities were set at 80% of active motor threshold (AMT). We used 2 trains of 600 pulses separated by 1 min (for a total of 1200 pulses) as opposed to the much longer standard protocols. This procedure may lead to stronger changes in the respective neural networks through growth and reorganization, resulting in longer-lasting behavioral effects [27,28]. It is important to note that the effects of Theta burst stimulation (TBS), particularly intermittent TBS, can last for up to 60 min or more after stimulation, depending on the protocol and individual variability [27,28].

Using individual MRI and a neuronavigation system, the cTBS was applied directly over the bilateral pre-SMA. Part of the supplementary motor cortex (SMC), the pre-SMA is thought to be involved in the more cognitive aspects of motor control, possibly in updating motor plans and in the learning of new motor sequences [29,30,31,32,33,34,35]. It is involved in higher-level aspects of motor behavior such as motor selection and inhibition. These properties are reflected in the pre-SMA’s distinct structural connectivity [36,37,38,39]. Anatomically, the pre-SMA is in the mesial part of the cerebral hemisphere and in the caudal part of the frontal lobe, rostral to the primary motor cortex and the SMA. It is illustrated in Figure 1b in a medial view of a human hemisphere. Whether reviewing nonhuman primate neurophysiology, human brain imaging, TMS or lesion studies to investigate the SMC, the consensus of research is that the more rostral end of the SMC, the pre-SMA, “is more likely to be active in more-complex or more-‘cognitive’ situations than the more caudal” SMA end [40]. These properties of the pre-SMA situate it at the critical junction between the current behavioral investigations and therapeutic interventions in GD patients.

### 2.3. Experimental Paradigm

Each GD patient, before and after the TMS or the SHAM protocol, performed a stop signal task (hereafter SST), whereas the non-treated group waited for the same amount of time between sessions while sitting. Participants viewed stimuli on a PC and the SST was generated with psychtoolbox 3 and custom MATLAB 2018b code [41]. Each trial of the SST started with a fixation cross for 350 ms followed by some arrows indicating left or right directions for 1400 ms (Figure 1) and then a fixation cross with a random time ranging from of 2000–3750 ms.

Participants were requested to press one of two buttons of a keyboard depending on the go stimuli direction (arrows). Intermittently and unpredictably, the participants were presented with a stop signal (red circle). In this case, participants were instructed to attempt to inhibit their response to the go signal (see Figure 1).

For the stop trials, the initial delay between the go and the stop signals was 300 ms. This delay was varied in a way that involves changing to suit the changing conditions, increasing or decreasing the stop signal delay of 34 ms. The lower limit was set to 100 ms.

A total of 250 trials divided into two sessions were administered to each participant, including an initial training phase. The first session was before the TMS protocol and the other one after the TMS stimulation. Each session was composed of 100 trials: 70% go, 20% stop and 10% rest trials, with the first session always preceded by a training session composed of a block of 50 trials, in order to ensure understanding of the instructions.

### 2.4. Statistical Methods

The variables used to evaluate the performance of the participants were go reaction times (RT), stop signal delay (SSD) and stop signal reaction times (SSRTs), computed with the mean method [42,43].

Two factors were considered as sources of variability: group (TMS group and non-treated group) and session (pre-TMS and post-TMS). Despite the name assigned, the non-treated group did not sit under the Magstim Rapid2 Stimulator with a SHAM coil, but instead waited during the interval between sessions. A two-factor mixed-model analysis of variance (group and session) was conducted on RT, SSD and SSRT. This analysis was chosen for its flexibility to designs that are not perfectly balanced (as in this case), considering the intrinsic (and uncontrolled) variability among the participants, everywhere treated as a random factor. Post-hoc Newman–Keuls comparisons were applied to interpret significant interactions.

## 3. Results

**Figure 2:**
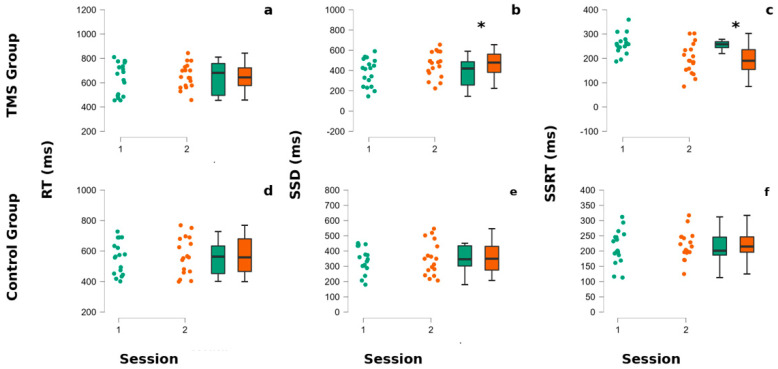
CTBS influences SSRT and SSD in gamblers. The superior part of the figure refers to the TMS group panels (**a**–**c**), whereas the lower part refers to the non-treated group’s performances (**d**–**f**). In each panel, the left portion represents dots indicating each participant’s performance, whereas in the right part of each panel are reported the associated box plots. The internal horizontal line of box plots indicates the mean of the participants’ performances. Colors code for the session of administration of the stop signal task (SST). The green color corresponds to the baseline (session 1), whereas the orange color corresponds to the second session (post-TMS for the TMS group and after the pause for the non-treated group). (**a**,**d**) The reaction times (RTs) of participants at the SST. (**b**,**e**) The stop Signal delay (SSD) of participants at the SST. (**c**,**f**) The stop signal reaction times (SSRTs) of participants at the SST. Asterisks indicate *p*-values < 0.05.

The task performance in patients with gambling disorder (*n* = 18) at baseline (pre-TMS session) revealed mean and standard error of reaction Times = 640 ± 27 ms; stop signal delay = 381 ± 26 ms and stop signal reaction times = 258 ± 12 ms. After the TMS treatment (post-TMS session) we observed mean and standard error of reaction times = 654 ± 27 ms; stop signal delay = 456 ± 26 and stop signal reaction times = 197 ± 12. As necessary to validate the performance at the SST, all participants had a probability of responding [p (respond|signal)] of between 0.25 and 0.75 [42,43,44]. Additional details of the behavioral performances (including the non-treated group) in the stop signal task are shown in Figure 2 and Table 1.

**Figure 3:**
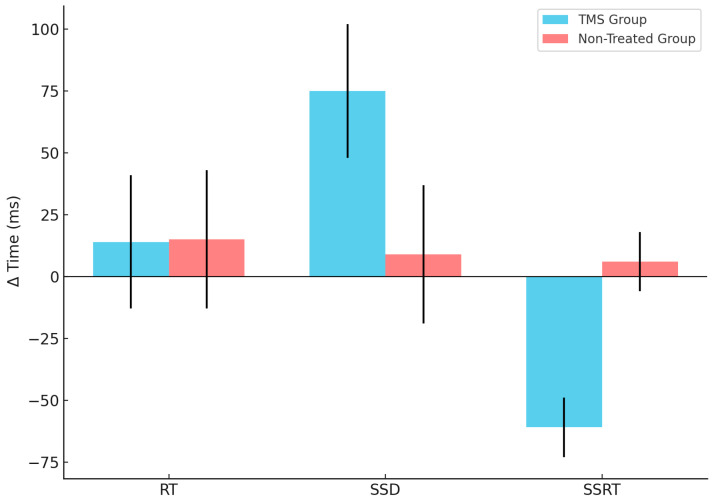
CTBS influences differently the Δ time of SSRT and SSD in the two groups. The blue refers to the TMS group, whereas the red refers to the non-treated group’s performances of the three parameters investigated—RT, SSD and SSRT (reported in X axis)—calculated as the Δ time (reported in Y axis) between session 2 (Post-TMS, when applicable) and session 1 (Baseline). The vertical lines represent the standard deviation.

A visual inspection (Figure 2 and Figure 3 and Table 1) shows that, in GD patients, the SSD and SSRT seem to be modified by the TMS stimulation. The statistical tests confirm this observation, showing a significant interaction between the group and session factors for SSD (F (1,33) = 16.99, *p* = 0.040) and SSRT (F (1,33) = 28.64, *p* = 0.000035). The post-hoc Newman–Keuls comparisons reveal that the TMS-treated group SSRT was significantly better after TMS application than at baseline (*p* = 0.0053) and with the first (0.030) and the second session of the non-treated group (*p* = 0.027), whereas the SSRT non-treated group did not significantly differ between session 1 and 2 (all *p* > 0.05). Regarding the SSD parameter, the post-hoc Newman–Keuls comparisons indicated that the TMS Group—at session 2—significantly improved with respect to baseline/first session (*p* = 0.05) and to the first (0.022) and second session (all *p* = 0.023) of the non-treated group.

All TMS-treated GD patients showed the same trend of responses. Conversely, the two SHAM patients showed an opposite trend of behavioral responses, as reported in Table 1.

## 4. Discussion

In this study, we found a significant improvement of the stop signal reaction time (SSRT) parameter in a group of subjects affected by GD (referred herein as “TMS group”) who received only one session of cTBS application as compared with the “untreated, healthy” non-treated group. This result was not enhanced due to any learning effect, given that there were no observed differences between pre and post task (i.e., stop signal task (SST)) session of the SSRT in the non-treated group.

Moreover, in the TMS-treated group of subjects, we found that in the second session of SST, the stop signal delay (SSD) differed significantly as compared with all other conditions. Finally, we did not find any significant differences in reaction times between TMS-treated and non-treated groups.

There appears to be only one study reporting that cTBS-treatment improves SST performance in healthy subjects after just one application of cTBS over the pre-SMA, as compared with a SHAM condition [18]. Our sample, however, may have missed this effect as it only included only two SHAM subjects.

To the best of our knowledge, the present investigation is the first one to report a reduction of SSRT after one session of cTBS over pre-SMA in a group of GD patients. As the SSRT in the second session did not change significantly between the two groups, one session of TMS-treatment may be enough to improve the inhibitory control of these participants. Whereas there is existing literature on SSD data in healthy subjects, we are not aware of similar data for GD subjects. It has been reported that an increase of SSD may suggest the implementation of the participants’ strategy to optimize their executive function performance [8]. In this case, it would be important to understand whether the degree of inhibitory demand imposed by the task has or has not been reduced. This condition can be assessed by observing whether there is a slowing in the reaction times to go signals. Individuals may strategically delay their behavioral responses and reaction times to go signals to increase stopping accuracy and SSD without a true increment in inhibitory efficiency [8,45,46,47,48,49]. Nonetheless, herein we found that the reaction times measured between the two groups for the go signal condition did not differ significantly. Because there was no adjustment in the reaction time, the reduction in SSD is even more interesting. Coupled with a decrease in the value of the SSRT, this condition suggests a strong enhancement in inhibitory efficiency. An interesting interpretation related to a difference in SSD and SSRT when reaction time does not differ within two sessions may be explored by temporally disentangling cognitive inhibitory control in proactive and reactive inhibition. According to Braver et al., [9] the proactive inhibition consists in a ‘top-down’ form of control to prepare the system for an upcoming event [9], whereas the reactive inhibition is a ‘bottom-up’ control implemented to stop an already initiated motor response [50]. These two processes can be separated depending on the time the action is withheld, both at the behavioral and the neural level [9,16,51,52].

Markedly, the improvement recorded after TMS treatment in SSRT and SSD without an effect on reaction times, or, in other terms, the degree of the participants’ commitment to the task, suggests an increase in the proactive inhibition of GD individuals more than on the reactive inhibitory process [8]. These cognitive measures suggest that TMS-treated GD participants become more efficient when keeping their system inhibited in response to upcoming events from the environment.

This behavioral improvement of proactive inhibitory efficiency intimates that TMS treatment modulates the inhibitory network associated with pre-SMA. According to our results, one single treatment of cTBS appears capable of influencing this inhibitory control. Nevertheless, additional work needs to dissociate the possibility that the observed effect may be due to the modulation of the neural network plasticity, or to a structural change in the fiber tract connecting the crucial brain regions of this inhibitory circuit. Based on the observations of the present study, of a longer SSD and a shorter SSRT, we could hypothesize that the most relevant part of the network underlying this effect would be the r-IFG. In a metanalysis, Gavazzi et al., [16] found that the r-IFG is the most crucial brain region in proactive inhibition. Additional work, however, is necessary to elucidate this process.

The current study has several limitations that need to be considered. First, we did not have a treatment-comparable SHAM group, but just two participants. Moreover, the two SHAM participants have inconsistent outcomes in SSRT. Notably, Table 1 shows that SHAM stimulation resulted in a change in SSRT for one subject and in SSD for both SHAM participants, with changes occurring in the same direction as in the TMS group. Although the sample size is too limited to draw firm conclusions, these results raise the possibility of unspecific effects or individual variability. Future studies should include a larger, well-matched SHAM-treated group to more definitively assess the specificity of cTBS effects. All of the participants of the TMS-treated group show an improvement in the SSRT parameter after the cTBS. Additionally, given that gambling disorder (GD) is a chronic and persistent condition, the use of a single cTBS session with follow-up limited to a single timepoint represents a significant limitation. The duration and clinical relevance of the observed effects remain unclear. While this design is appropriate for a preliminary investigation, further studies should incorporate repeated sessions and longer follow-up periods to better assess the sustainability and therapeutic potential of cTBS in GD. Finally, our results are consistent with Obeso et al., [18], who showed no improvement in SSRT in healthy subjects of the SHAM group with the coil over the pre-SMA but an improvement of SSRT when cTBS was applied to the pre-SMA.

Future studies using T1, T2, DTI and fMRI will be needed to determine if cTBS over the pre-SMA influences the structure or function of the inhibitory brain network in GD and healthy controls, and which of the neural circuits demonstrate changes in neuroplasticity.

## 5. Conclusions

This study reports, for the first time, that after one cTBS session localized over the pre-SMA, GD patients demonstrates an improvement of the key parameters of the SST. Despite the small number of subjects and fewer members of the SHAM group, the current data make a strong case for a real effect of the cTBS treatment on a central brain circuit dysfunction in GD.

From a clinical perspective, previous research on GD patients has shown that stimulating cTBS over pre-SMA in patients with GD produces, according to psychometrical assessment, beneficial effects in the reduction of GD symptomatology [19,20]. Our results would suggest that the clinical improvements reported might be mainly ascribed to an ameliorated proactive inhibitory efficiency, rather than to the cognitive shifting ability or a generally improved decision-making capacity [53,54].

Finally, to address the practical question of whether cTBS can be employed as a standard treatment for GD, some practical aspects need to be considered. Based on our results, we can conclude that further research is necessary to determine whether the observed effects are sustained over time or require repeated stimulation sessions. Secondly, TMS treatment is relatively expensive, as it requires specialized equipment and trained personnel. Therefore, while our findings are promising, the broader clinical adoption of TMS for GD will depend on overcoming these practical challenges.

## Figures and Tables

**Table 1:** Behavioral performances. Values are in milliseconds.

SSRT	SSD	RT	TMS Group (n = 18)
258 ± 12	381 ± 27	640 ± 27	Baseline
197 ± 12	456 ± 27	654 ± 27	Post-TMS
−61 ± 12	75 ± 27	14 ± 27	Δ Time
**SSRT**	**SSD**	**RT**	**Non-treated group (n = 17)**
212 ± 12	342 ± 28	555 ± 28	Baseline
218 ± 12	351 ± 28	570 ± 28	Post-Pause
6 ± 12	9 ± 28	15 ± 28	Δ Time
**SSRT**	**SSD**	**RT**	**SHAM TMS—case 1**
328	311	639	Baseline
352	390	742	Post-TMS
24	79	103	Δ Time
**SSRT**	**SSD**	**RT**	**SHAM TMS—case 2**
458	402	860	Baseline
359	449	808	Post-TMS
−99	47	−52	Δ Time

## Data Availability

The data presented in this study are available on request from the corresponding author. Data is unavailable due to privacy and ethical restrictions.
